# Linc-KILH potentiates Notch1 signaling through inhibiting KRT19 phosphorylation and promotes the malignancy of hepatocellular carcinoma

**DOI:** 10.7150/ijbs.52279

**Published:** 2021-02-08

**Authors:** Xudong Zhang, Xiaoliang Xu, Zechuan Zhang, Cailin Xue, Zhijun Kong, Siyuan Wu, Xiao Yun, Yue Fu, Chunfu Zhu, Xihu Qin

**Affiliations:** 1 The Affiliated Changzhou NO.2 People's Hospital of Nanjing Medical University, 29 XingLongXiang Road, Changzhou, Jiangsu 213000, P.R. China.; 2 School of medicine, Southeast University, Nanjing, China.; 3 Department of Hepatobiliary Surgery of Nanjing Drum Tower Hospital, Nanjing Medical University, Nanjing, China.

**Keywords:** hepatocellular carcinoma, microvascular invasion, long noncoding RNAs

## Abstract

Long noncoding RNAs (LncRNAs) are emerging as crucial regulators in the pathophysiological process of various tumors, including HCC. Here, we identify a novel lncRNA Linc-KILH (KRT19 interacting long noncoding RNA in hepatocellular carcinoma), which is significantly up-regulated in HCC tissues and positively correlated with larger tumor size, severer microvascular invasion, more intrahepatic metastasis and decreased survival of HCC patients. Silence of Linc-KILH remarkably inhibited the proliferation and metastasis abilities of KRT19-positive HCC cells* in vitro* and *in vivo*. Mechanistically, Linc-KILH interacts with KRT19 and then inhibits the phosphorylation of KRT19 on Ser35, thereby, enhancing the translocation of KRT19 from cytoplasm to membrane in KRT19 positive HCC cells. Additionally, we validated that KRT19 interacts with β-catenin but not RAC1 in HCC cells. Linc-KILH enhanced the interaction between β-catenin and KRT19 in cytoplasm and promoted the nuclear translocation of β-catenin in HCC cells. Furthermore, Linc-KILH could enhance the promoting function of KRT19 on Notch1 signaling with the existence of KRT19 in HCC cells. Collectively, we revealed that Linc-KILH exerts a vital function in KRT19 positive HCC progression and may likely be developed into an effective therapeutic target for HCC.

## Introduction

As a highly lethal cancer, it is estimated that hepatocellular carcinoma (HCC) causes almost half a million deaths annually in East Asia and South Africa 1. Although continuous advances in HCC treatment (for example, surgical resection, liver transplantation, radiation therapy, targeted chemotherapy and molecular-targeted therapy) have been developed, the long-term survival rate of HCC patients still remains low 2, 3. Therefore, there is an urgent need to further comprehensively explore the underlying molecular mechanisms of HCC and identify novel prognostic and therapeutic targets.

KRT19 (Cytokeratin 19), one of the smallest members of the acidic type I cytokeratin family proteins (KRTs), has been reported as widely detectable tumor maker in various cancers, including HCC 4-7. Several studies have revealed that KRT19 can function as a marker of poor prognosis in HCC 8-10. For instance, knockdown of KRT19 suppress the progression of HCC and its stem cell properties through regulating TGFβ/Smad and Notch1 signaling pathways 5. Additionally, a recent study revealed that activation of PDGFR alpha/laminin B1/KRT19 signaling pathway promotes the progression of HCC through developing microvascular invasion, metastasis and early recurrence in HCC 11. As a cytoplasmic intermediate filament protein, KRT19 can regulate diverse properties in various cancers through interacting with a variety of signal transduction molecules. Recent studies shown that KRT19 could function as a differential modulator of Wnt/β-Catenin/Notch signaling and resulted in opposing regulation of cancer properties in breast and colon cancers 6, 7. Thus, extensive molecular researches on the specific functional regulation of KRT19 in HCC need to be further elucidated.

Long noncoding RNAs (LncRNAs) are traditionally identified as transcripts longer than 200nt and with no or limited protein coding ability 12. Accumulating researches have revealed that LncRNAs are regularly dysregulated and closely correlated with the development and outcome of various cancers, including HCC 13, 14. Up to now, the researches of liver cancer related LncRNAs is still in the development stage, more efforts should be put into the exploration of functional LncRNAs in HCC. Among the various mechanisms of LncRNAs, one of the most interesting way is to participate in the regulation of downstream signaling pathways through the interaction with binding proteins 15-18. For instance, a recent study revealed a lncRNA interacting with STAT3 and then regulating its phosphorylation, thus modulated STAT3 signal transduction in dendritic cells 19. Here, we proposed that further identification and functional elucidation of KRT19 associated LncRNAs could provide new insights into KRT19-mediated tumor promotion in HCC. However, whether or not LncRNAs could participate in KRT19 associated signaling pathways in HCC has not been studied.

In our present research, we attempt to explore the precise regulation mechanism of KRT19 on Wnt/Notch signaling pathway in HCC and whether or not there were LncRNAs involving in this regulation. We identified a KRT19 interacting lncRNA Linc-KILH, which specifically binds to KRT19 and restrains its phosphorylation, thus inhibiting the membrane translocation of KRT19 and then enhancing the combining capacity between KRT19 and β-catenin, which eventually stabilizes and enhances the promoting function of KRT19 on Notch1 pathway in HCC. Moreover, Linc-KILH was overexpressed in HCC tissues, and its deficiency inhibited HCC proliferation and metastasis *in vitro* and *in vivo*. As a consequence, Linc-KILH enhanced the malignant phenotypes of hepatoma cells. Our findings further validated the mechanism of LncRNAs which play an oncogenic role through modulating the phosphorylation status of its interacting protein.

## Materials and methods

### Patient samples and cell lines

116 paired pathologically diagnosed HCC samples (including tumor tissues and matched non-tumor liver tissues) were obtained between May 2010 and October 2014 at The Affiliated Changzhou NO.2 People's Hospital of Nanjing Medical University (Changzhou, Jiangsu, China). Written consent approving the usage of tissues in our study were obtained from each patient. The histopathological diagnoses of HCC were conducted by three experienced pathologists independently. This study was approved by the ethnic committee of the Affiliated Changzhou NO.2 People's Hospital of Nanjing Medical University ([2017]KY013-01). Human HCC cell lines (SMMC-7721, Huh7, HepG2, MHCC-97H, MHCC-97L, Hep3B) and the human normal liver L02 cell line were obtained from KeyGen (Nanjing KeyGen Biotech Co., Ltd., Jiangsu, China). All cells used in our study were cultured in Dulbecco's modified Eagle's medium (DMEM, Invitrogen Life Technologies, Carlsbad, CA, USA) supplemented with 10% fetal bovine serum (FBS, Gibco, Carlsbad, CA, USA) and 1% streptomycin/penicillin at 37°C in a humidified environment consisting of 95% air and 5% CO2.

### Real-time quantitative PCR

To isolate total RNA from cultured cells or tissues, we used TRIZOL reagent (Invitrogen, CA, USA) according to the manufacturer's instructions. Reverse transcription and real-time PCR were then conducted using complementary NDA (cDNA) Synthesis SuperMix (TransGen Biotech, China) and SYBR Green real-time PCR kit (Toyobo, Japan). The primers used in our present study were listed in Table S1. GAPDH served as the internal control.

### Ectopic expression and gene silencing

To effectively knockdown Linc-KILH and KRT19 expression in HCC cells, we designed and constructed corresponding shRNAs and matched negative control shRNAs, and then cloned them into lentivirus vector GV248 (Gene, Shanghai, China), respectively. All the shRNA oligos used in our study are listed in Table S2. In addition, to overexpress Linc-KILH, we subcloned the sequence of Linc-KILH into the lentiviral vector GV367 (Gene, Shanghai, China). All vectors used in our study were labeled with luciferase, and the transfection procedures were manipulated in accordance with the manufacturer's instructions.

### Western blotting and antibodies

Briefly, protein from cells and tissues were isolated using RIPA lysis buffer and then separated on SDS-PAGE gels and transferred to PVDF membranes. And then, the membranes were blocked with 5% skim milk in TBST, followed by incubated with the indicated primary antibodies overnight at 4 °C and then with secondary horseradish peroxidase (HRP)-conjugated secondary antibodies. Antibodies used in our study are as followed: KRT19 (Abcam, ab52625), β-catenin (Abcam, ab32572), RAC1 (Abcam, ab33186), c-Jun (Abcam, ab32137), Notch1 (Proteintech, 20687-1-AP), Jagged1 (Proteintech, 66890-1-Ig), RBP-Jk (Abcam, ab25949), Hes1 (CST, 11988), CDK2 (Proteintech, 10122-1-AP), CyclinD1 (Proteintech, 60186-1-Ig), Vimentin (Abcam, ab137321), E-cadherin (CST, 3195), GAPDH (Proteintech, 60004-1-Ig). Protein expression signals were detected by a Chemiluminescent Imaging System (Thermo Scientific, IL, USA).

### Cell growth, migration and invasion assays

The growth curve of HCC cells was examined by using Cell Counting Kit-8 (CCK-8) (Dojindo, Japan) and EdU Assay Kit (Guangzhou RIBOBIO, Guangzhou, China) in accordance with the manufacturer's instructions. 5 × 10^4^ cells supplemented with 200 μl serum-free DMEM were added into the upper chamber with or without Matrigel (BD Biosciences, San Jose, CA) for invasion or migration invasion assays. 800μl DMEM supplemented with 20% FBS was added into the lower chambers. After incubation at 37 °C for 24 hours, the cells migrated or invaded to the lower side of the membrane were stained with 0.1% crystal violet solution, and then imaged and calculated by using a microscope (Olympus, Japan).

### *In vivo* experiments

To further examine the biological function of Linc-KILH on tumor growth *in vivo*, tumor subcutaneous xenograft assay was conducted by using six-week-old male nude mice (BALB/c background). A total of 5×10^6^ Huh7 and Hep3B cells transfected with indicated vectors were subcutaneously into the left or the right flanks of mice. 5 weeks later, mice were sacrificed and the xenograft were isolated from mice and the volume of each tumor were calculated (length × width^2^ × 0.5). For lung metastasis model, MHCC-97H and Hep3B cells transfected with indicated vectors were suspended in 200 μl PBS and then intravenous injected through the tail. 6 weeks later, mice were sacrificed and the lung metastasis situation were detected by the IVIS Lumina II system (Caliper Life Sciences, MA, USA). The lung of each mice was stained by hematoxylin and eosin and the number of metastasis was calculated. All animal experiments were performed in accordance with the Institutional Animal Care and Use Committee guidelines of the Affiliated Changzhou NO.2 People's Hospital of Nanjing Medical University.

### Immunohistochemistry (IHC) assays

Immunohistochemistry assays were conducted by using paraffin-embedded sections of tissue samples. KRT19 expression was validated in tumor tissues obtained from HCC patients by using anti-KRT19 antibody (Abcam, ab52625). The positive percentage of Ki67 and PCNA was examined in mice xenograft tumor tissues by using anti-Ki67 antibody (Abcam, ab15580) and anti-PCNA antibody (Abcam, ab92552). The tissues were incubated with primary antibodies overnight at 4 °C and then with HRP-conjugated secondary antibody. Immunohistochemical staining were visualized using DAB. A digitalized microscope camera (Olympus, Japan) was used to visualize and calculate the stained sections.

### Subcellular fractionation assays

To separate the cytoplasm and cytomembrane protein, we used the plasma membrane protein isolation kits (Invent Biotechnologies, Eden Prairie, MN, USA, SM005) in accordance with the manufacturer's instructions. And then, the nuclear fraction was extracted using the nuclear extraction buffer (20 mM HEPES (pH 7.9), 400 mM NaCl, 1 mM EDTA, 1 mM EGTA, 1 mM DTT, and 1 mM PMSF) with 10 min agitation at 4 °C and subsequently centrifuged at 13,000 rpm for 10 min at 4 °C.

### RNA pulldown assay

RNA pull-down was performed as previously described 20. Briefly, biotin-labeled Linc-KILH and control RNA were produced *in vitro* using Biotin RNA Labeling Mix (Roche) and T7 RNA polymerase (Promega) and then were purified using an RNeasey Mini Kit (Qiagen, Valencia, CA, USA) after treatment with RNase-free DNase I (Roche). Three μg biotinylated RNAs were mixed with proteins extracted from Huh7 cells, followed by targeting RNAs with streptavidin beads (Millipore, Bedford, MA, USA). Finally, the retrieved proteins were washed with a RIPA buffer and subjected into SDS-PAGE for separation, followed by Western blotting.

### RIP and RNA-seq assays

RIP and RNA-seq assays were performed as previously otherwhere reported 21. Briefly, to conduct RIP assay, we used the Magna RIP™ RNA Binding Protein Immunoprecipitation Kit (Millipore, Massachusetts, USA) in accordance with the manufacturer's instructions. And then, the RNAs gotten from RIP assay were directly applied for the synthesis of ds-cDNA. Next, the ds-cDNA was attached to the adapters and sequenced using Illumina Genome Analyzer (Novogene). Cufflinks was used to quantify the levels of RNAs and then normalized to FPKM. In addition, reads aligned to non-polyA transcripts were excluded. To compute the fold enrichment for RIP, we normalized the FPKM value of each transcript to the average of all transcripts.

### Statistical analysis

All data were analyzed using the appropriate statistical analysis methods with SPSS software (version 19.0, IBM, Armonk, NY), and the data are expressed as the means ± SD. Student's t-test was used to analyze the differences between 2 groups, while one-way ANOVA was applied for multiple comparisons. Overall survival (OS) and recurrence-free survival (RFS) were assessed using Kaplan-Meier method and the significance was determined by log-rank test. *P*-value < 0.05 was considered as statistical significance.

## Results

### KRT19 binds a lincRNA Linc-KILH in HCC cells

**Figure 1:**
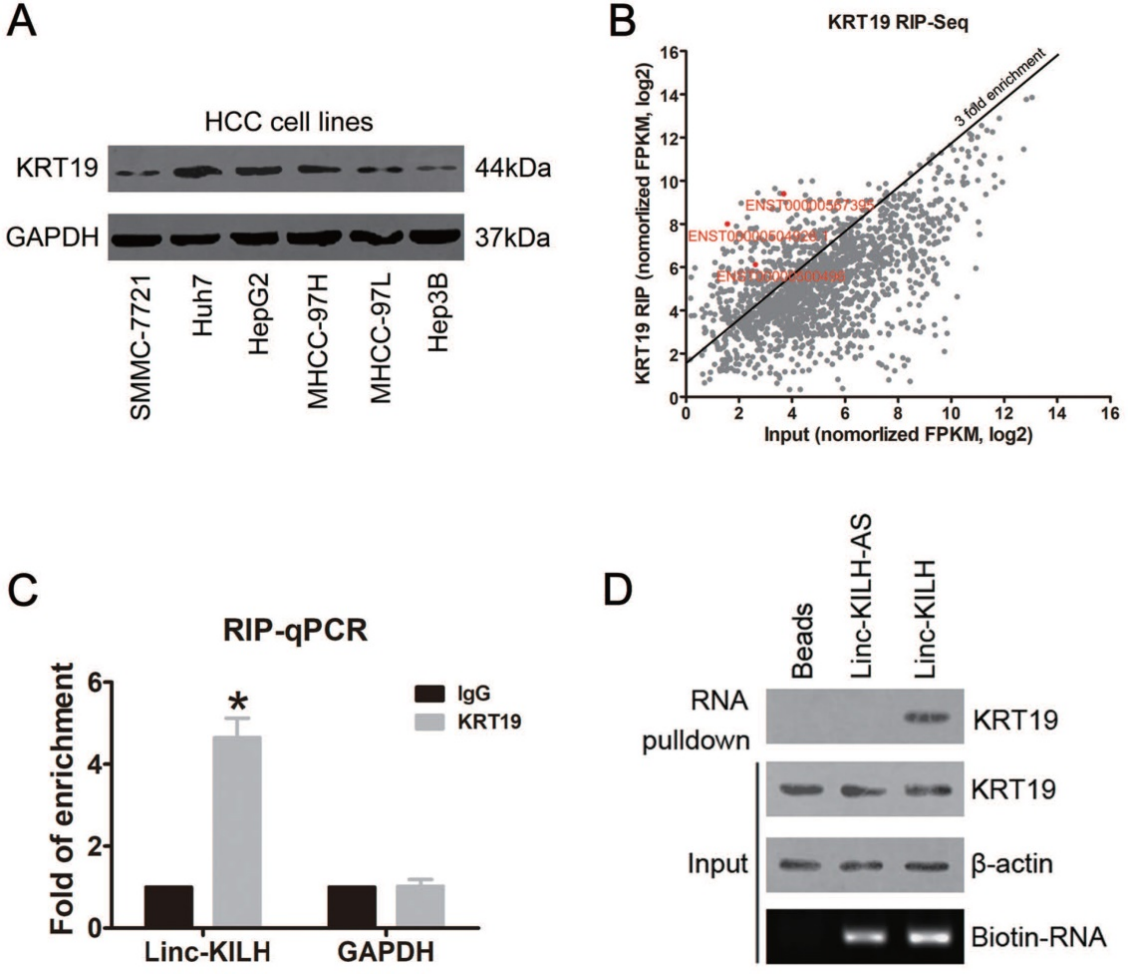
KRT19 binds a lincRNA Linc-KILH in HCC cells. (A) The expression level of KRT19 in HCC cell lines was detected by immunoblotting. (B) Huh7 cells were subjected to RIP assays with a KRT19 antibody or isotype-matched control IgG, RIP-Seq analysis showing average-normalized, log2 transformed FPKM value of KRT19-RIP and input. (C) The amount of Linc-KILH in the precipitates was analyzed by quantitative real-time PCR analysis. RNA enrichment indicates the RNA levels of Linc-KILH or GAPDH in the anti-KRT19 precipitates relative to those in the IgG precipitates. D, Biotin-labeled Linc-KILH and Linc-KILH-AS were incubated with Huh7 cell lysate, and KRT19 interaction was confirmed by Western blotting. *P < 0.05.

KRT19 was reported to express in various cancers, including HCC, but 10%-28% of hepatocellular carcinomas were reported to express KRT19, and those HCC patients with KRT19 positive were found to display a more invasive phenotype and poorer prognosis 5, 22, 23. Here, we checked the protein level of KRT19 in 116 HCC tissue samples by immunohistochemistry staining (IHC), and KRT19 positive expression was found in 26 cases (26/116, 22.4%) (Figure S1A). In addition, we examined the mRNA level of KRT19 in the HCC tissue mentioned above by RT-qPCR (Figure S1B). Also, KRT19 protein level was evaluated in HCC cell lines (Figure 1A). Similarly, we got a differential expression pattern of KRT19 in HCC cells and Huh7 cells showed the highest expression.

The differential expression of KRT19 in HCC tissues and cell lines and its function in mediating Notch1 signaling indicates that there may exist other regulators which could regulate its signal adjusting function 5-7. Here, we proposed to identify KRT19 interacting RNAs, especially long noncoding RNAs. RNA immunoprecipitation and sequencing (RIP-Seq) was conducted in KRT19 highest expression Huh7 cells. Using > 3-fold enrichment and FPKM value > 2 in input as a cutoff, we identified a total of 256 transcripts bound by KRT19, excitingly, three LncRNAs were found bound by KRT19. In addition, we found that ENST00000504928.1 showed the richest content in IP sample compared with input (Fold of enrichment ~80) (Figure 1B) (Table S3). Next, we examined the levels of these 3 LncRNAs in 20 pairs of randomly selected, paired tumor and corresponding non-tumor liver tissues by RT-qPCR, and found that only ENST00000504928.1 was remarkably overexpressed in HCC tumor tissues compared with the corresponding non-tumor liver tissues (Figure S2). Therefore, we named it as KRT19 interacting long noncoding RNA in hepatocellular carcinoma (Linc-KILH). Next, we confirmed the binding association between KRT19 and Linc-KILH by RIP-qRCR, RNA pulldown and Western blotting analysis (Figure 1C and 1D).

### Linc-KILH expression is upregulated in HCC tissues and correlated with prognosis of patients with HCC

**Figure 2:**
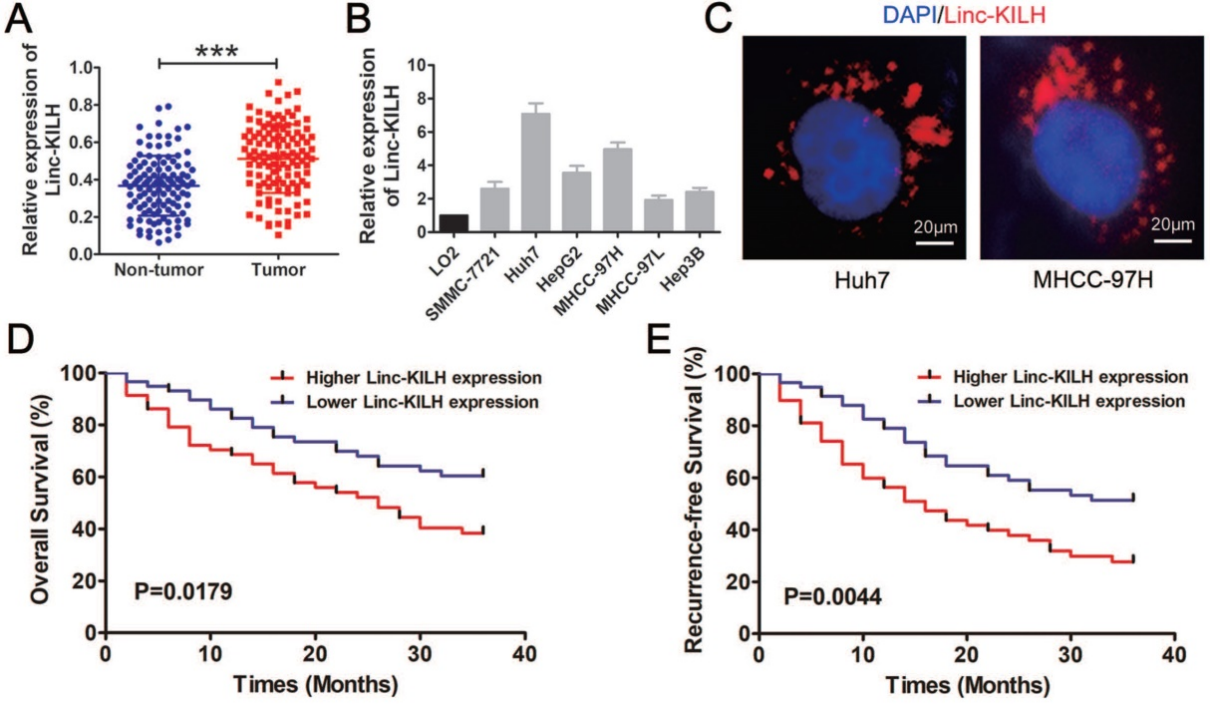
Linc-KILH is up-regulated in HCC tissues and correlated with the prognosis of patients with HCC.** (**A) The expression level of Linc-KILH was detected by RT-qPCR in HCC tumor tissues and adjacent non-tumor liver tissues. (B) Relative expression of Linc-KILH in human L02 hepatocytes and HCC cell lines was detected by RT-qPCR. (C) The subcellular location of Linc-KILH was analyzed through fluorescence *in situ* hybridization (FISH) (original magnification ×400). (D) The overall survival and recurrence-free survival rates of 116 HCC patients were compared between higher Linc-KILH and lower Linc-KILH groups. ***P < 0.001.

To further validate the increase of Linc-KILH, its expression was further analyzed by RT-qPCR in 116 pairs of HCC tumor and non-tumor tissues. We revealed that Linc-KLIH was obviously overexpressed in tumorous tissues compared with the corresponding non-tumor liver tissues (Figure 2A). In addition, we found that the expression of Linc-KILH in KRT19 positive HCC tissues was significantly higher than that in KRT19 negative HCC tissues (Figure S1C). Next, we investigated the expression pattern of Linc-KILH in HCC cells, and validated that Linc-KILH was also up-regulated in most HCC cell lines compared with normal liver cell L02 (Figure 2B). And then, its coding potential was examined by using Coding Potential Calculator and CPAT 24, 25, and results from both program indicated that Linc-KILH has no coding ability (Figure S3A and S3B). Intracellular location of Linc-KILH was visualized in Huh7 and MHCC-97H cells by RNA fluorescence *in situ* hybridization (FISH) assays, and it mainly locates in the cytoplasm of HCC cells (Figure 2C). To assess whether the aberrant expression of Linc-KILH and KRT19 are related to HCC progression, the clinical information was collected and analyzed in HCC patients mentioned above. As shown in Table 1, we divided all 116 patients into Linc-KILH high and low groups according to the median value of Linc-KILH, and KRT19 positive and negative groups according to the results of KRT19 IHC staining. Statistical analysis shown that both high Linc-KLIH and positive KRT19 expression was obviously associated with increased tumor size (*P* = 0.001 for Linc-KILH; *P* = 0.001 for KRT19), severer microvascular invasion (*P* = 0.009 for Linc-KILH; *P* = 0.008 for KRT19), and more intrahepatic metastasis (*P* = 0.010 for Linc-KILH; *P* = 0.025 for KRT19). Furthermore, Kaplan-Meier and log-rank test analyses revealed that higher Linc-KILH expression was obviously associated with decreased overall survival (OS) and recurrence-free survival (RFS) rates (*P* = 0.0179 for OS and *P* = 0.0044 for RFS) (Figure 2D and 2E). In addition, KRT19 positive expression in HCC tissues obviously correlated with decreased overall survival (OS) and recurrence-free survival (RFS) rates (P = 0.0363 for OS and P = 0.0027 for RFS) (Figure S1D and S1E).

### Linc-KILH enhances the proliferation, migration and invasion abilities of KRT19 positive HCC cells *in vitro*

**Figure 3:**
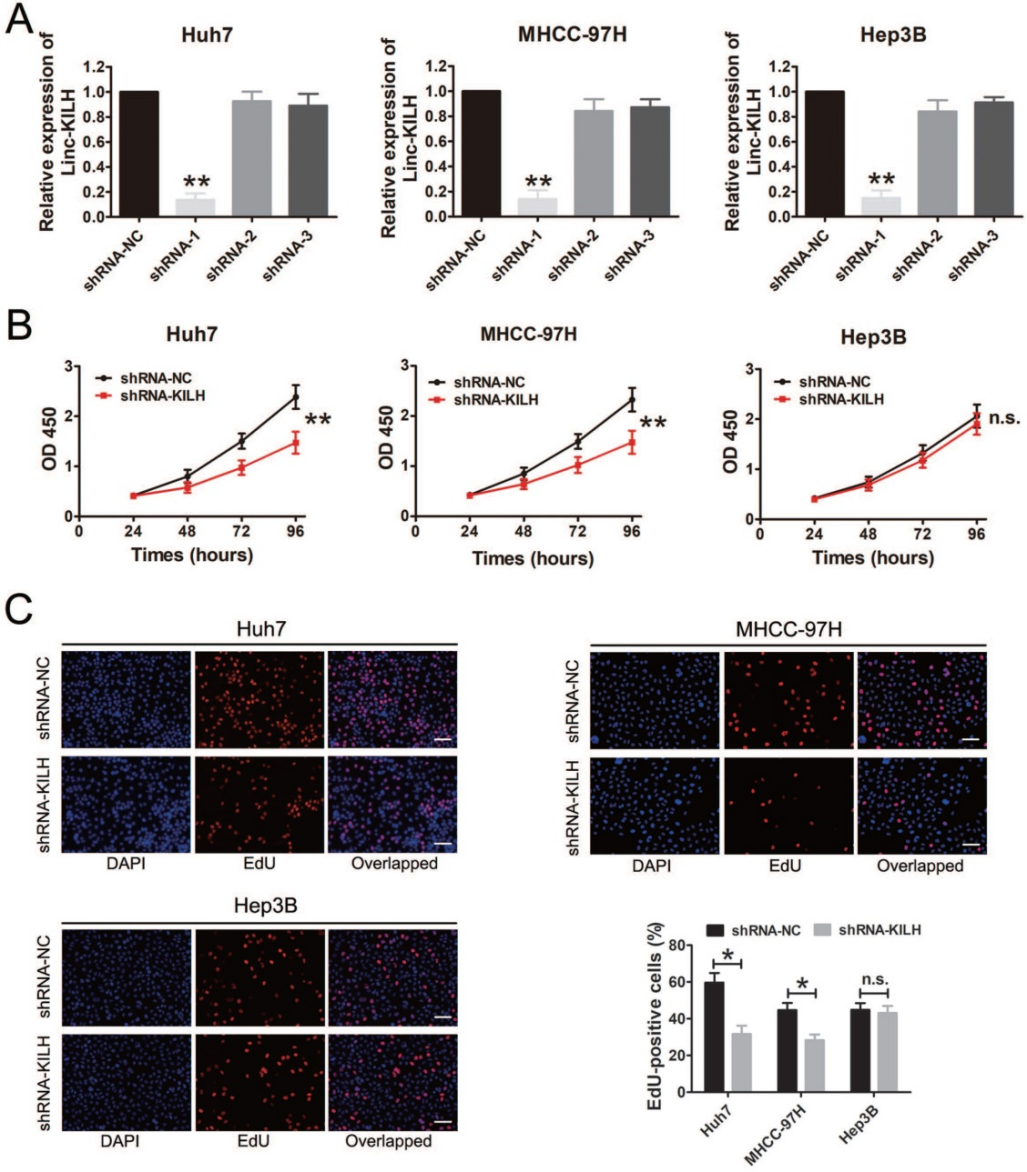
Linc-KILH promotes HCC cell proliferation *in vitro*. (A) Gene silence of Linc-KILH using Linc-KILH shRNAs in Huh7, MHCC-97H and Hep3B cells was conducted and the transfection efficiency was validated by RT-qPCR. (B) Cell proliferation assays for Huh7, MHCC-97H and Hep3B cells infected with the lentivirus silencing Linc-KILH or the control using Cell Counting Kit-8 assay. (C) EdU staining was used to detected the proliferation abilities of HCC cells knockdown of Linc-KILH (original magnification ×100). Each experiment was performed in triplicate and the results are shown as the means ± SD. *P < 0.05; **P < 0.01.

**Figure 4:**
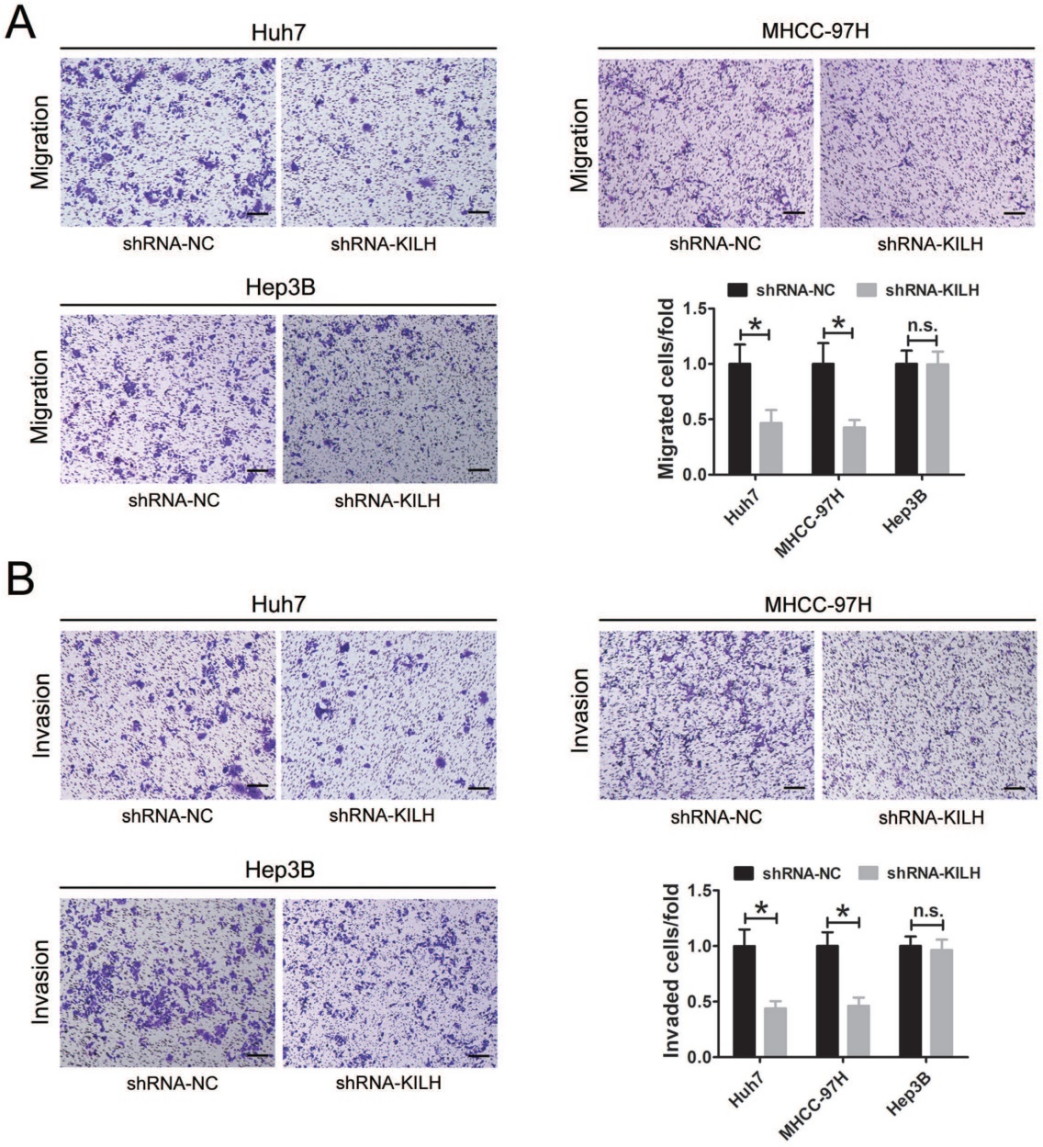
Linc-KILH enhances the migration and invasion abilities of HCC cells *in vitro.* (A and B) Transwell assays were conducted to examined the migration and invasion abilities of Huh7, MHCC-97H and Hep3B cells transfected with Linc-KILH shRNA (original magnification ×100). Each experiment was performed in triplicate and the results are shown as the means ± SD. *P < 0.05.

According to the expression level of Linc-KLIH and KRT19 in HCC cell lines, we defined Huh7 and MHCC-97H as KRT19-positive cells, Hep3B as KRT19-negative cells to further explore whether or not Linc-KILH participate in the physiology function of KRT19 in HCC cells. Three independent shRNA plasmids were generated and transfected in Huh7, MHCC-97H and Hep3B cells, as shown in Figure 3A, only shRNA-1 exhibited the most knockdown efficiency and it was applied to investigate the biological function of Linc-KILH on HCC cells. CCK8 assay was used to determine the proliferation ability of HCC cells, knockdown of Linc-KILH prominently suppressed the proliferation of Huh7 and MHCC-97H cells, whereas, no change was gotten in Hep3B cells (Figure 3B). In addition, EdU (5-ethynyl-2′-deoxyuridine) assay was conducted and the same results were gotten (Figure 3C). In addition, we conducted transwell assays to investigate HCC cells migration and invasion abilities and found that silence of Linc-KILH obviously inhibited the migration and invasion abilities in KRT19-positive Huh7 and MHCC-97H cells while no significant change was obtained in KRT19-negative Hep3B cells (Figure 4A and 4B). Based on the functional experiments above, we confirmed that Linc-KILH significantly enhanced the proliferation, migration and invasion abilities of KRT19 positive HCC cells *in vitro*.

### Linc-KILH facilitates KRT19 positive HCC cells growth and metastasis *in vivo*

**Figure 5:**
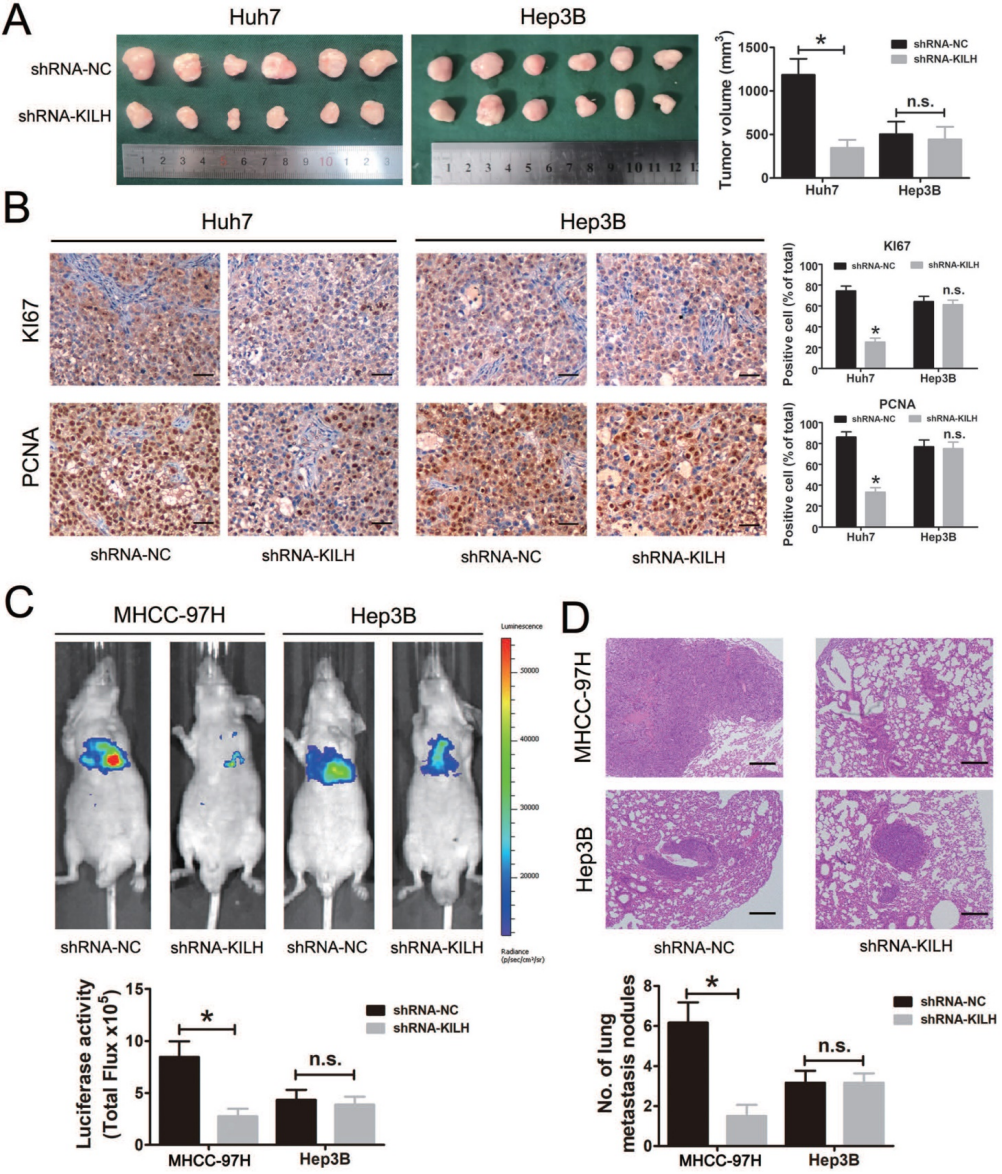
Linc-KILH facilitates HCC growth and metastasis *in vivo*. (A) Huh7 and Hep3B cells knockdown of Linc-KILH and the control cells were subcutaneously injected into 6-week-old BALB/c nude mice, 5 weeks later, the mice were sacrificed and the tumor size was measured. (B) The percentage of Ki-67 and PCNA positive staining cells was detected by immunohistochemistry in the tumor tissues from the subcutaneous xenograft model (original magnification ×100). (C) A lung metastasis model was established in which mice were injected with HCC cells (5 × 10^6^ cells suspended in 200 µL PBS) through the tail vein and the lung metastasis was investigated respectively using the IVIS Lumina II system. Representative images of a mouse in each group were presented. (D) All the results of lung colonization were validated by the histological examination (H&E) (original magnification ×100). Metastatic tumors with volumes >2mm^3^ were identified and compared in each group. Experiments were performed in triplicate independently and the results are shown as the means ± SD. *P < 0.05.

We then determined the biological function of Linc-KILH on HCC growth and metastasis *in vivo*. Here, mouse subcutaneous xenograft models were established, we injected Huh7 and Hep3B cells with stable silencing of Linc-KILH subcutaneously in nude mice. Tumor xenografts derived from Linc-KILH silencing Huh7 cells had smaller mean volumes compared with control cells, while no difference was gotten in Hep3B cells (Figure 5A). Next, we detected the positive rates of Ki67 and PCNA in the tumor tissues from the subcutaneous xenograft models by immunohistochemistry. As shown in Figure 5B, Linc-KILH silencing decreased the positive percentage of Ki-67 and PCNA staining cells in Huh7 cells, whereas no alteration was gained in Hep3B cells. To further explore the biological function of Linc-KILH on HCC metastasis *in vivo*, we conducted mice lung metastasis models by tail vein injecting Linc-KILH stably silenced MHCC-97H and Hep3B cells which were labeled with firefly luciferase. After injection for 6 weeks, mice were sacrificed and metastases were evaluated. Obviously, the bioluminescent signals were significantly reduced in the Linc-KILH knockdown MHCC-97H cells, while no significant change was observed in Hep3B cells (Figure 5C). H&E staining further confirmed a reduction of metastatic lesions in the lung of mice that received Linc-KILH-knockdown MHCC-97 cells (Figure 5D). In summary, our present data suggest that Linc-KILH enhances the growth and metastasis abilities of KRT19 positive HCC *in vivo*.

### Linc-KILH inhibits the phosphorylation of KRT19 to potentiate Notch1 signaling in HCC

**Figure 6:**
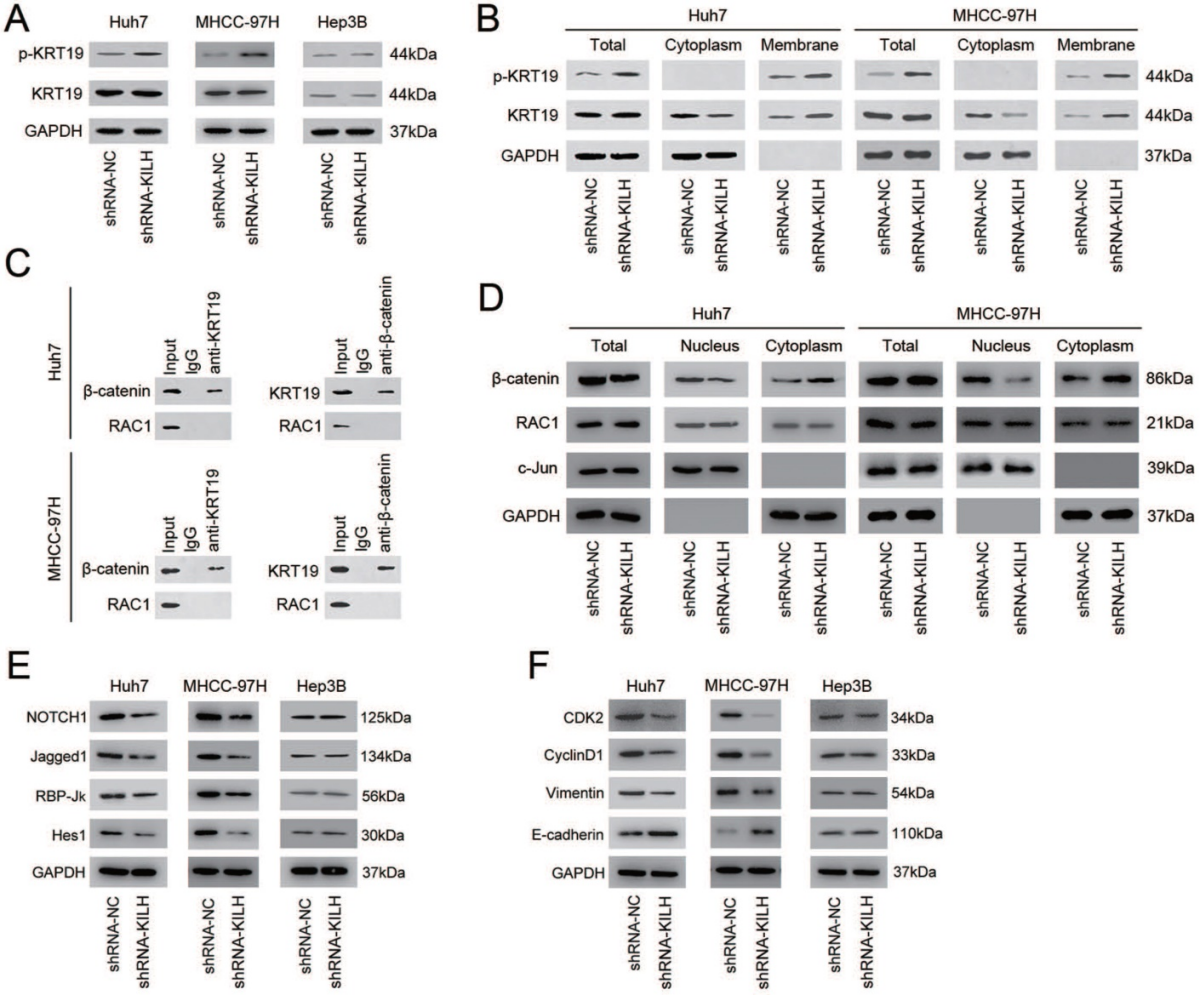
Linc-KILH inhibits the phosphorylation of KRT19 to potentiate Notch1 signaling in HCC. (A) The expression levels of total and phosphorylated KRT19 on Ser35 site in Huh7, MHCC-97H and Hep3B cells upon Linc-KILH alteration were examined by western blotting. (B) The subcellular location and the content of the phosphorylated and total KRT19 were detected by western blotting in KRT19 positive Huh7 and MHCC-97H cells. (C) Co-immunoprecipitation (Co-IP) was performed using Protein A/G Sepharose and antibodies specific for KRT19, β-catenin, and RAC1, or normal IgG in KRT19 positive Huh7 and MHCC-97H cells. Cell lysates were analyzed by Western blotting. (D) Cell fractionation assay for β-catenin and RAC1 analyzed by Western blotting; actin and c-Jun were used as the cytoplasmic and nuclear markers, respectively, in KRT19 positive Huh7 and MHCC-97H cells. (E) The variation of the expression of the members of Nothc1 signaling pathway in Linc-KILH-silenced cells was determined by western blotting. (F) The expression levels of cell proliferation and EMT transformation related genes were examined by western blotting in HCC cells upon Linc-KILH silence.

Studies have revealed that one of the most prevalent mechanisms underlying the interaction between LncRNAs and their binding proteins was adjusting the phosphorylation status of proteins 19, 26, 27. The phosphorylation status of KRT19 on Ser35 has been recognized as the most important regulation pattern of KRT19 in various biological behaviors, including tumors 28-30. Since Linc-KILH was identified bound to KRT19 and could influence the biological properties of HCC cells with the existence of KRT19, we proposed that it might participate in the regulation of the phosphorylation of KRT19. To confirm this hypothesis, we detected the protein content of the phosphorylated KRT19 on Ser35 and the total KRT19 by immunoblotting in HCC cells. As shown in Figure 6A, silence of Linc-KILH in KRT19-positive Huh7 and MHCC-97H cells obviously increased the phosphorylation status but not the total content of KRT19, whereas no alteration was gained in KRT19-negative Hep3B cells. Studies have reported that once KRT19 was phosphorylated on Ser35, it would remodel its form from filamentous to granulous mold and translocate from the cytoplasm to cell membrane. Since Linc-KILH could adjust KRT19 phosphorylation, we suspect that it might promote the translocation of KRT19 in HCC cells. We extracted and isolated proteins from cell membrane and cytoplasm with a commercial kit and examined the protein content of the phosphorylated and total KRT19 respectively. As exhibited in Figure 6B, KRT19 phosphorylated on Ser35 mainly located in the cell membrane and knockdown of Linc-KILH strengthened the translocation of KRT19 from cytoplasm to cell membrane in Huh7 and MHCC-97H cells.

Studies investigating the regulation function of KRT19 on Wnt/Notch signaling in tumors have gained an opposing result, and the key point underlying this opposing regulation was whether KRT19 could bind to RAC1 in cytoplasm of tumor cells 6, 7. In mammary cancer, KRT19 was reported to interact with β-catenin/RAC1 complex and then upregulate NUMB expression, thus suppressing Notch signaling 6. However, in colon cancer, KRT19 was validated to interact with β-catenin but not with RAC1 and then enhance the transcription function of LEF/TCF, thus promoting Notch signaling 7. Now that researches have validated the promoting function of KRT19 on Notch1 signaling in HCC cells, we proposed that the function of KRT19 in HCC might be similar with that in colon cancer 5, 31. Co-IP assays was conducted to explore this hypothesis, and the results proved that KRT19 only interacted with β-catenin, but not RAC1 in HCC cells (Figure 6C). The interaction of KRT19 and β-catenin in cytoplasm stabilized and enhanced the translocation of β-catenin from cytoplasm to nucleus and then enhanced the Notch signaling. Here, we validated that Linc-KILH could inhibited the phosphorylation of KRT19 and strengthened its cytoplasmic expression. Next, we aimed to examine the function of Linc-KILH on the translocation of β-catenin in HCC cells. We found that knockdown of Linc-KILH in Huh7 and MHCC-97H cells attenuated nuclear translocation of β-catenin, while there was no change of the nuclear RAC1 content upon Linc-KILH silence (Figure 6D). Therefore, we elucidated that KRT19 interacted with β-catenin and Linc-KILH could regulated β-catenin nuclear import but not RAC1 in HCC cells.

Next, we confirmed the regulation effect of Linc-KILH on Notch1 signaling in HCC cells by immunoblotting. We found that silence of Linc-KILH suppressed Notch1 signaling in KRT19-positive Huh7 and MHCC-97H ells, and no difference was observed in KRT19-negative cells (Figure 6E). Notch1 signaling has been reported as a cancer promoting pathway in HCC through enhancing tumor cell proliferation, migration, invasion and EMT transformation. Here, we detected some related markers (CDK2, CyclinD1, Vimentin, E-cadherin) by immunoblotting and found that knockdown of Linc-KILH in Huh7 and MHCC-97H cells reduced the protein levels of CDK2, CyclinD1 and Vimentin, but increased E-cadherin protein level, while no change was obtained in Hep3B cells (Figure 6F). Taken together, we validated that Linc-KILH could inhibit the phosphorylation of KRT19 on Ser35 and then enhanced its translocation from cytoplasm to membrane thus inhibiting β-catenin nuclear import, and finally potentiated Notch1 signaling in HCC.

### KRT19 is essential for the tumor promoting function of Linc-KILH in HCC

**Figure 7:**
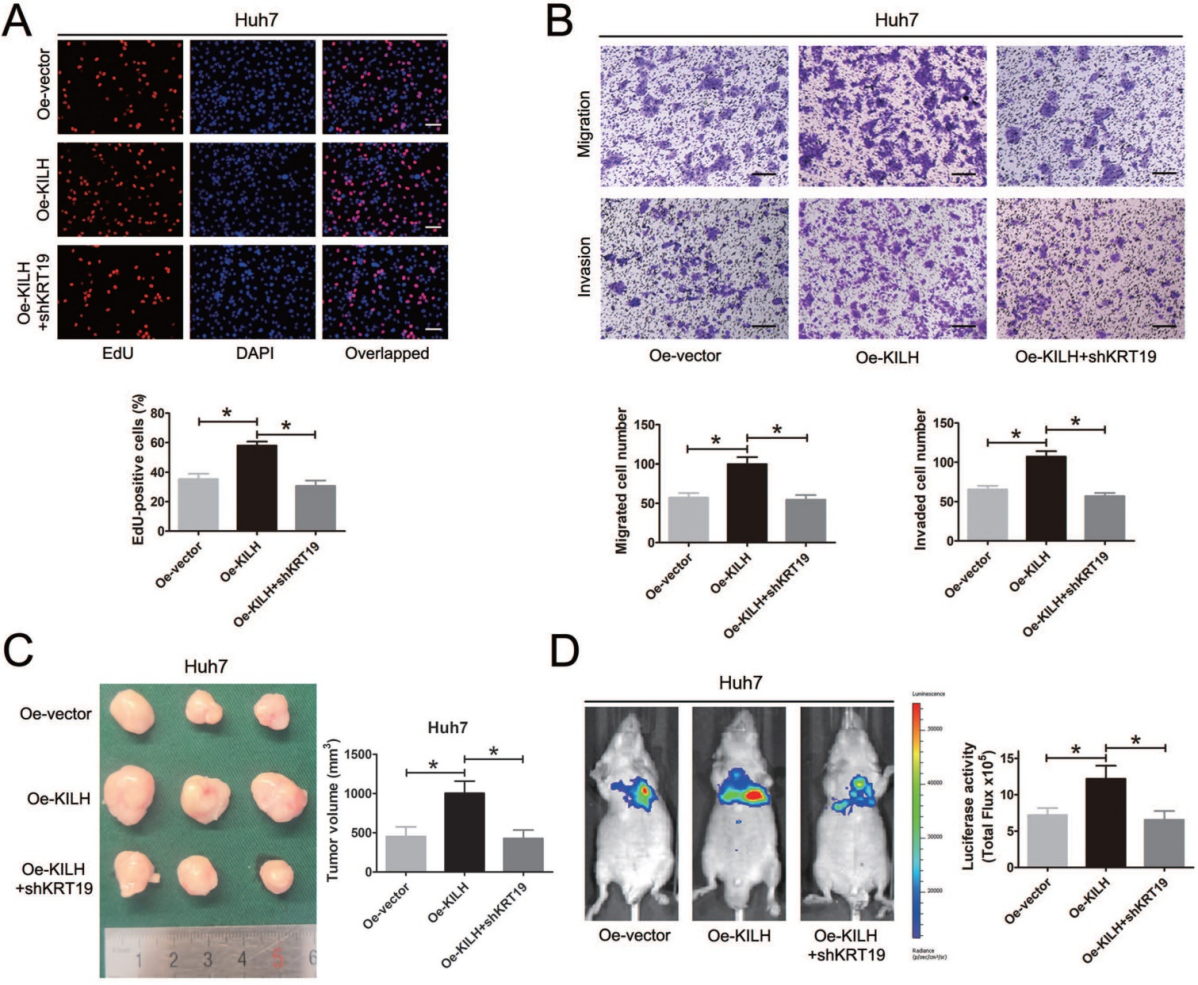
KRT19 is essential for the tumor promoting function of Linc-KILH in HCC. (A) EdU staining was used to detected the proliferation abilities of HCC cells when Linc-KILH was overexpressed with and without KRT19 silence in Huh7 cells (original magnification ×100). (B) Transwell assays were conducted to examined the migration and invasion abilities of Linc-KILH overexpressed Huh7 cells upon KRT19 silence (original magnification ×100). (C) Images and volume of xenografts established by subcutaneous transplantation with Linc-KILH overexpression and KRT19 knockdown were exhibited and calculated. (D) Representative images of the lung metastasis model and the luciferase activities were measured by IVIS imaging system. Each experiment was performed in triplicate and the results are shown as the means ± SD. *P < 0.05.

*In vitro* and *in vivo* functional experiments in Huh7, MHCC-97H and Hep3B cells revealed that the cancer promoting function of Linc-KILH on HCC cells was dependent on KRT19 expression. To further validate the necessity of KRT19 for Linc-KILH, we conducted a series of rescue experiments. Firstly, we overexpressed Linc-KILH in Huh7 cells and the transfection efficiency was examined by RT-qPCR (Figure S4A). And then, we silenced KRT19 in Linc-KILH overexpressed Huh7 cells, and the knockdown efficiency was examined by RT-qPCR and immunoblotting respectively (Fig. S4B and S4C). EdU assays was conducted to examine the proliferation ability of HCC cells, overexpression of Linc-KILH significantly enhanced the growth of Huh7 cells, whereas knockdown of KRT19 practically abolished the enhancement effect of Linc-KILH (Figure 7A). Similarly, transwell assays demonstrated that silence of KRT19 almost eliminated the promoting function of Linc-KILH on the migration and invasion abilities of HCC cells (Figure 7B). As shown in Figure 7C, the *in vivo* subcutaneous experiments certified that overexpression of Linc-KILH obviously strengthened the growth capacity of Huh7 cells, and knockdown of KRT19 removed the effect of Linc-KILH. Results from the mice metastasis model also confirmed this phenomenon (Figure 7D). Collectively, these data further demonstrated that the cancer promoting function of Linc-KILH was dependent on the presence of KRT19 in HCC cells.

## Discussion

As far as we know, this is the first discovery of long noncoding RNA bound to KRT19 (Linc-KILH) and explores its biological function in HCC. We demonstrated Linc-KILH was significantly upregulated in HCC tissues compared with the corresponding non-tumor tissues in our study cohort. Correlation analysis revealed that high Linc-KILH expression was positively correlated with advanced tumor progression and poorer prognosis of HCC patients. Functionally, silence of Linc-KILH significantly repressed the growth and metastasis abilities of KRT19 positive HCC cells *in vitro* and *in vivo*. Therefore, our study suggested that Linc-KILH could function as a crucial factor for the regulation of HCC progression.

With a large number of LncRNAs been fully explored, many aspects of their biological and physiological functions and the underlying mechanisms have been validated 13, 15, 32, 33. One of the most prevalent functional patterns of LncRNAs is forming complexes with proteins and then regulation the status of the binding proteins through various posttranslational modifications 34-38. For instance, TSLNC8 could function as a tumor suppressor through physically interacting with STAT3 and modulating its phosphorylation on Tyr705 and Ser727 sites 38. Lnc-EGFR was reported to specifically bind to EGFR and then restrained its ubiquitination, thus stimulating Treg cells differentiation in HCC 34. KRT19 has been demonstrated as a tumor promoting factor in various tumors, including HCC, whereas the detailed underlying mechanisms are still sciolistic 5-7. In our present study, we proposed to concentrate on KRT19 interacting RNAs, especially LncRNAs. We conducted RIP-Seq analysis in KRT19 highest expression Huh7 cells and successfully identified a long noncoding RNA Linc-KILH interacting with KRT19 in HCC. Followed RIP-qPCR, RNA pulldown and immunoblotting assays reconfirmed the physically interacting between Linc-KILH and KRT19 in HCC cells. Furthermore, we demonstrated that Linc-KILH could inhibit the phosphorylation status of KRT19 on Ser35 site. Studies have revealed that once KRT19 was phosphorylated on Ser35, it would transform from filamentous form to granulous form and translocate from cytoplasm to cell membrane. Here, we demonstrated that Linc-KILH participated in this translocation regulation and promoted the membrane translocation of KRT19.

An opposing concept has been acquired on the regulation function of KRT19 on Wnt/Notch signaling in tumors 7. KRT19 could interact with β-catenin/RAC1 complex and then upregulate NUMB expression, thus suppressing Notch signaling in mammary tumor 6. However, in colon cancer, KRT19 was reported to interact with β-catenin, but not RAC1, and then enhanced the transcription function of LEF/TCF, thus promoting Notch signaling 7. The critical factor on the basis of this opposing regulation is whether KRT19 could bind to RAC1 in the cytoplasm of tumor cells. However, KRT19 was validated to promote Nothc1 signaling in HCC, thus, these discoveries encouraged us to explore the detailed roles of KRT19 on Notch1 signaling in HCC. Here, we present strong evidence that KRT19 interact with β-catenin but not RAC1 in HCC cells. Linc-KILH enhanced the interaction of β-catenin of KRT19 in cytoplasm and promoted the nuclear translocation of β-catenin in HCC cells. Signaling detecting revealed that Linc-KILH could enhance the promoting function of KRT19 on Notch1 signaling with the existence of KRT19 in HCC cells. *In vitro* and *in vivo* functional experiments in Huh7, MHCC-97H and Hep3B cells revealed that the cancer promoting function of Linc-KILH on HCC cells was dependent on KRT19 expression. Furthermore, we conducted a series of rescue experiments, and the results further validated the necessity of KRT19 for the promoting function of Linc-KILH in HCC cells.

In conclusion, we validate Linc-KILH as a tumor promoting lncRNA, which is up-regulated in HCC tissues and could act as a molecular regulator of KRT19 to heighten Notch1 signaling, and in turn enhances the growth and metastasis abilities of KRT19 positive HCC cells, consequently boosting the development of HCC. We also detailed the mechanism of Linc-KILH on the regulation of KRT19 and β-catenin subcellular localization. Our findings indicate that Linc-KILH exerts a vital effect in KRT19 positive HCC development and may likely be developed into an effective therapeutic target for HCC.

## Supplementary Material

Supplementary figures and tables.

## Figures and Tables

**Table 1:** Correlation between Linc-KILH and KRT19 expression and clinicopathological characteristics of HCC patients

Characteristics	Linc-KILH		*P* value	KRT19		*P* value
	High	Low		Positive	Negative	
All cases	58	58		26	90	
**Age, years**			0.850			0.833
<60	35	34		15	54	
≥60	23	24		11	36	
**Gender**			0.342			0.590
Male	49	45		22	72	
Female	9	13		4	18	
**AFP**			0.678			0.554
≤20U/L	17	15		6	26	
>20U/L	41	43		20	64	
**HBsAg**			0.226			0.304
Positive	50	45		23	72	
Negative	8	13		3	18	
**Liver cirrhosis**			0.696			0.155
Yes	39	37		20	56	
No	19	21		6	34	
**Tumor size**			**0.001**			**0.001**
≤5 cm	19	36		5	50	
>5 cm	39	22		21	40	
**Tumor number**			0.243			0.316
Single	44	49		19	74	
Multiple	14	9		7	16	
**Microvascular invasion**			**0.009**			**0.008**
Yes	25	12		14	23	
No	33	46		12	67	
**Intrahepatic metastasis**			**0.010**			**0.025**
Yes	12	3		7	8	
No	46	55		19	82	
**Edmondson**			0.342			0.590
I-II	9	13		4	18	
III-IV	49	45		22	72	

Total data from 116 HCC patients were analyzed. For the expression of Linc-KILH, median expression level was used as the cutoff. Data were analyzed by chi-squared test. *P*-value in bold indicates statistically significant.
