# Linc-KILH potentiates Notch1 signaling through inhibiting KRT19 phosphorylation and promotes the malignancy of hepatocellular carcinoma: Erratum

**DOI:** 10.7150/ijbs.96688

**Published:** 2024-04-05

**Authors:** Xudong Zhang, Xiaoliang Xu, Zechuan Zhang, Cailin Xue, Zhijun Kong, Siyuan Wu, Xiao Yun, Yue Fu, Chunfu Zhu, Xihu Qin

**Affiliations:** 1 The Affiliated Changzhou NO.2 People's Hospital of Nanjing Medical University, 29 XingLongXiang Road, Changzhou, Jiangsu 213000, P.R. China.; 2 School of medicine, Southeast University, Nanjing, China.; 3 Department of Hepatobiliary Surgery of Nanjing Drum Tower Hospital, Nanjing Medical University, Nanjing, China.

In our paper, the authors noticed an error in Figure 5. In Figure 5D, we mistakenly used the typical HE staining photograph intended for shRNA-KILH in 97H cells from the shRNA-KILH Hep3B folder. The correct version of Figure 5 is provided below. The rest of the data and the conclusions originally reported in the paper remain unchanged. We apologize for any inconvenience that this error may have caused.

## Figures and Tables

**Figure 5:**
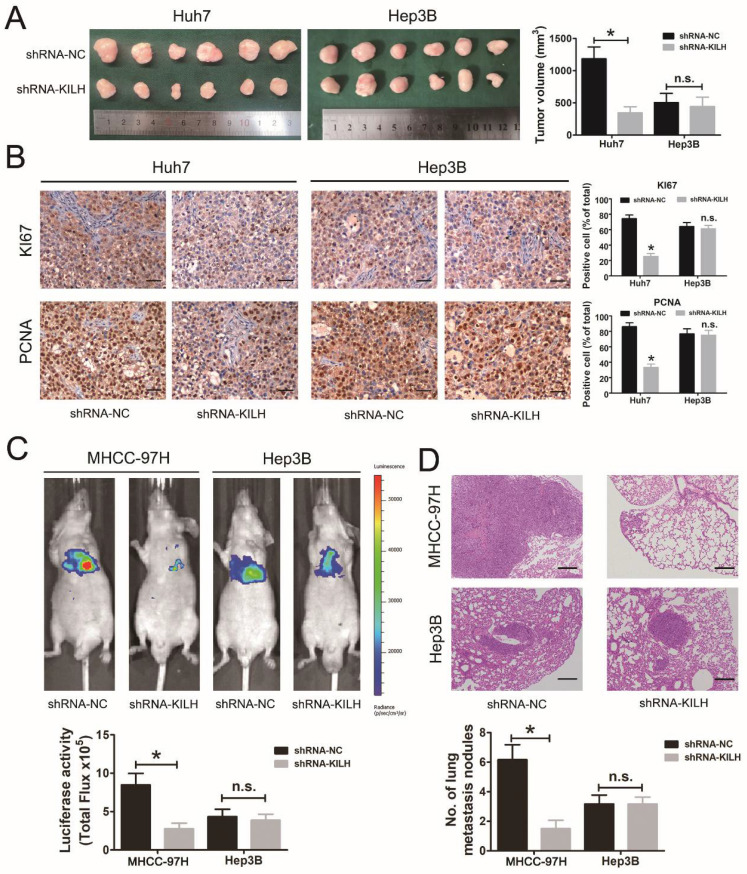
Correct image.

