# Supplementary material for: Linc-KILH potentiates Notch1 signaling through inhibiting KRT19 phosphorylation and promotes the malignancy of hepatocellular carcinoma
Source: Int J Biol Sci. 2021 Feb 8;17(3):768–80. doi: 10.7150/ijbs.52279 (PMC7975697; doi:10.7150/ijbs.52279)
Supplement: Supplementary file 1 — Supplementary figures and tables. [file ijbsv17p0768s1.pdf]

Supplementary Information for:

Linc-KILH potentiates Notch1 signaling through inhibiting KRT19 phosphorylation and promotes the malignancy of hepatocellular carcinoma

Xudong Zhang[1, +], Xiaoliang Xu[2, 3, +], Zechuan Zhang[3, +], Cailin Xue[1], Zhijun Kong[1], Siyuan, Wu[1], Xiao Yun[1], Yue Fu[1], Chunfu Zhu[1, *], Xihu Qin[1, *]

[1]The Affiliated Changzhou NO.2 People's Hospital of Nanjing Medical University, 29 XingLongXiang Road, Changzhou, Jiangsu 213000, P.R. China.

[2]School of medicine, Southeast University, Nanjing, China.

[3]Department of Hepatobiliary Surgery of Nanjing Drum Tower Hospital, Nanjing Medical University, Nanjing, China.

[*]Corresponding authors.

[+]These authors contributed equally to this work.

Contents:

1. **Supplementary Figures**

2. **Supplementary Tables**

1. Supplementary Figures:

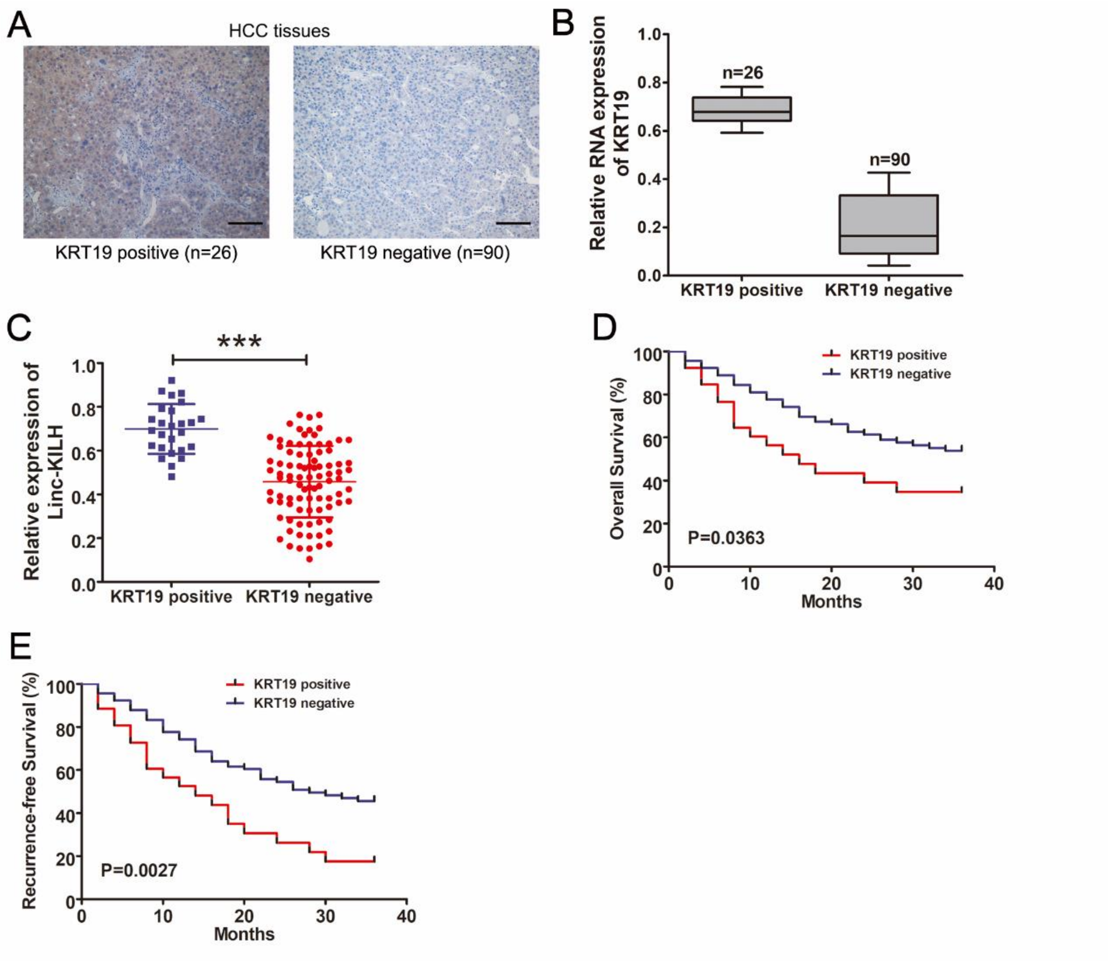

Figure S1. The protein and mRNA expression levels of KRT19 in HCC tissues. (A) Immunochemistry (IHC) was used to detect the protein expression of KRT19 in 116 HCC tissues, and KRT19 positive expression was found in 26 cases (26/116, 22.4%), representative staining of KRT19 positive and negative tissues was shown (original magnification ×100). (B) The mRNA level of KRT19 was examined in the tissues mentioned above. (C) The expression of Linc-KILH in KRT19 positive HCC tissues was significantly higher than that in KRT19 negative HCC tissues. (D and E) In our study cohort, we found that KRT19 positive expression in HCC tissues obviously correlated with decreased overall survival (OS) and recurrence-free survival (RFS) rates (P = 0.0363 for OS and P = 0.0027 for RFS). ***$P < 0.001$.

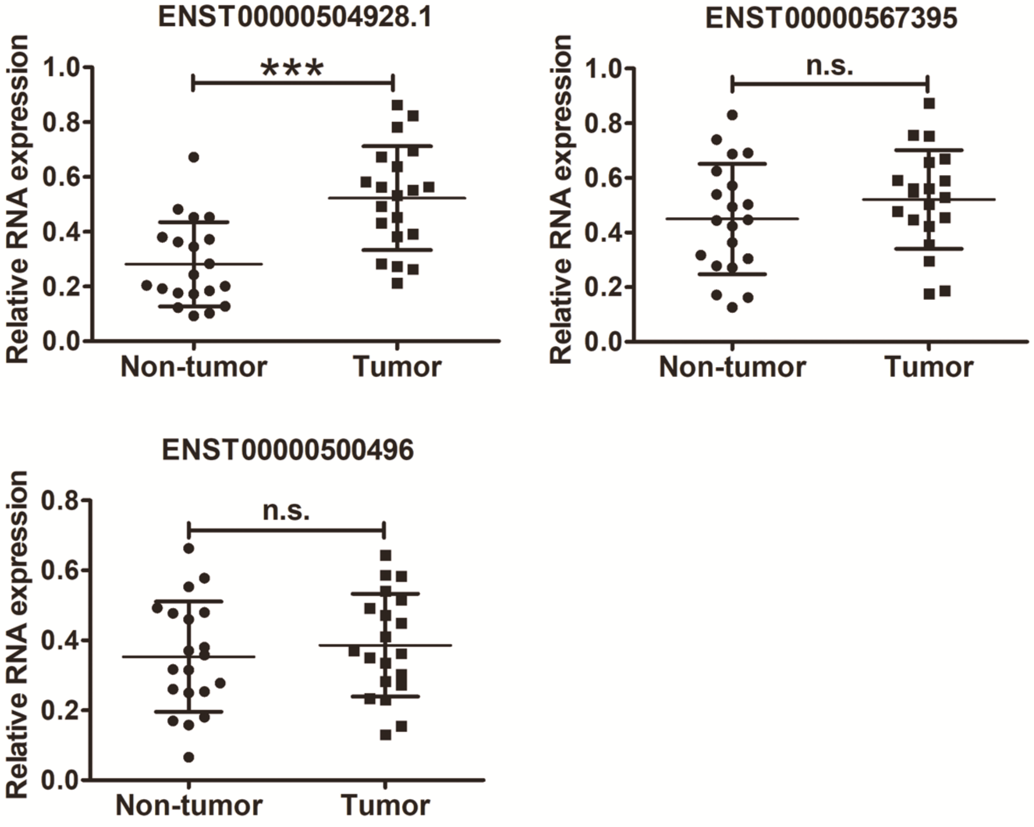

Figure S2. Detection of the expression of lncRNAs identified in the RIP-seq assay. The expression levels of the 3 lncRNAs selected in the KRT19 RIP-seq assay was examined in a cohort of 20 paired HCC tissues and corresponding non-tumor liver tissues. ***$P < 0.001$.

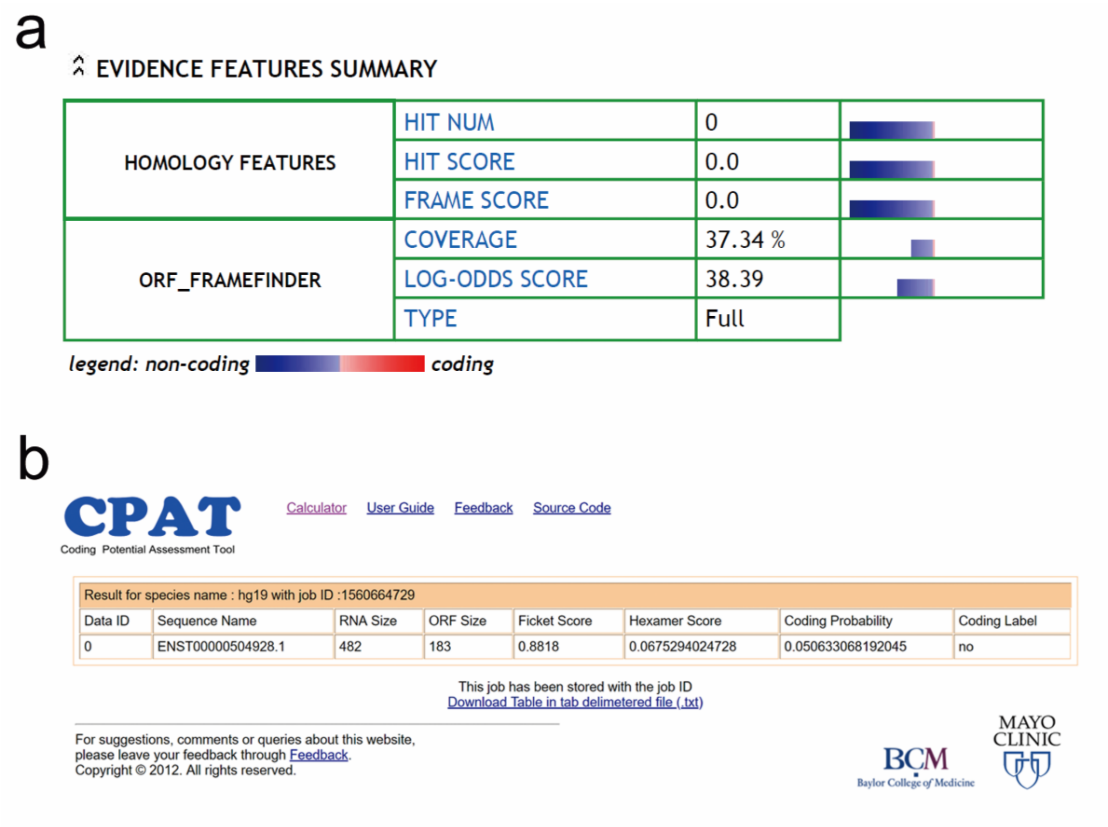

Figure S3. Evaluation of the coding potential of Linc-KILH. (A) Coding Potential Calculator was used to evaluate the coding potential of Linc-KILH. (B) CPAT also was used to detect the coding potential of Linc-KILH.

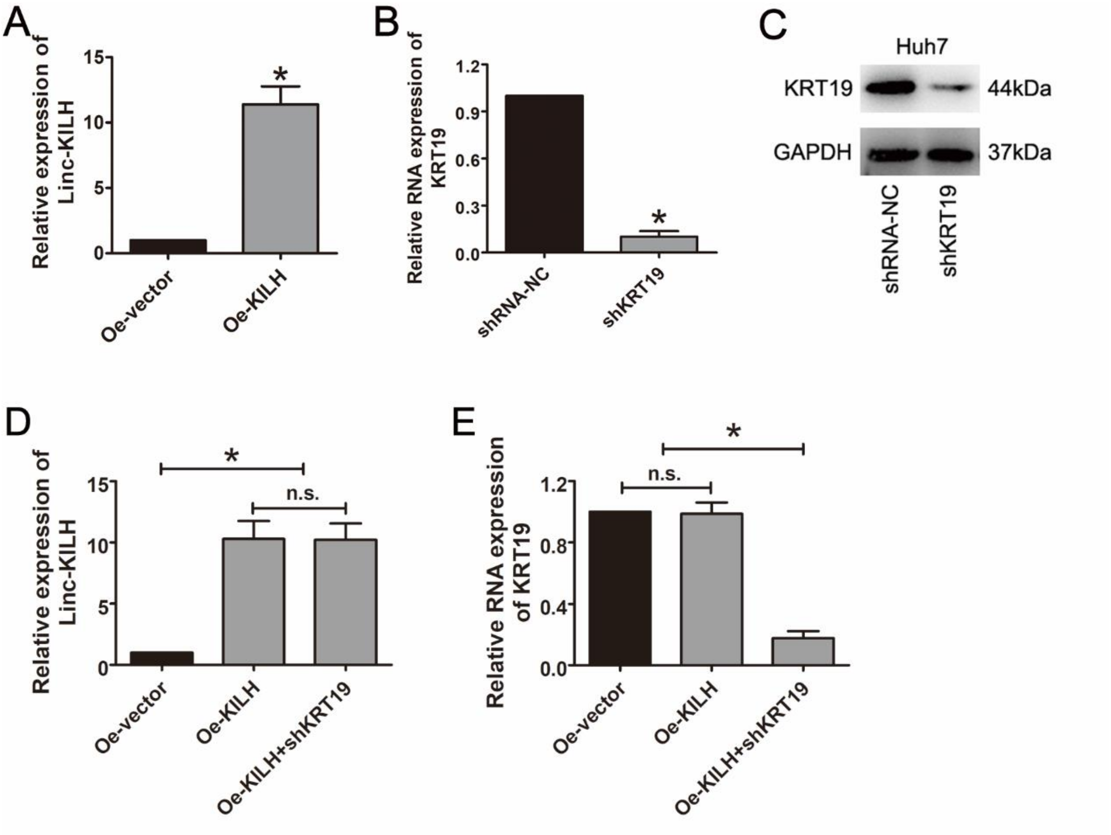

Figure S4. Detection of the transfection efficiency. (A) The overexpression efficiency of Linc-KILH in Huh7 cells was detected by RT-qPCR compared with the control group. (B and C) The knockdown efficiency of KRT in Huh7 cells was detected by RT-qPCR and western blot compared with the control group. (D and E) We detected the level of Linc-KILH and KRT19 in the tumors of the two mice model of figure 7C-D after the Linc-KILH overexpression and KRT19 knockdown. *P<0.05.

2. Supplementary Tables:

Table S1. Primers for Quantitative RT- PCR and methylation.

Gene name	Primer	Sequence
KRT19	Forward Primer	GGGTGTGTGTCTAACCTAATGG
	Reverse Primer	GGCCAGAATACTCTCCTTCAACT
Linc-KILH	Forward Primer	TGGTGGAAGGCAAAGTAGGA
	Reverse Primer	TGCTCTAGTTTCAGCAGTCAGTG
GAPDH	Forward Primer	GGAGCGAGATCCCTCCAAAAT
	Reverse Primer	GGCTGTTGTCATACTTCTCATGG
ENST00000567395	Forward Primer	ATGGGCCCTCACTAGACAGA
	Reverse Primer	CTTTGCCTTTGCCACTCTCT
ENST00000500496	Forward Primer	GGGAGGGCTGAGTCACTTG
	Reverse Primer	GCTGGGCTGCAGATCTTT

Table S2. Summary of shRNA Oligos.

Gene	Oligo Sequence
Linc-KILH shRNA 1	GACCTAACTAGAGGGAGAG
Linc-KILH shRNA 2	GAGCTTCAACTTCTCCTCT
Linc-KILH shRNA 3	GGAATCATTGCATGACCTG
KRT19 shRNA	GAAGACACACTGGCAGAAA

Table S3. Three candidate lncRNAs identified by KRT19 RIP-seq in Huh7 cells.

LncRNA	Chromosome	LncRNA-start	LncRNA-end	Enrichment Fold
ENST00000504928.1	Chr6	53564297	53583333	82.3
ENST00000567395	Chr16	23020337	23023611	51.6
ENST00000500496	Chr15	90660234	90664967	10.5
